# Artificial Intelligence-Based Diagnosis of Cardiac and Related Diseases

**DOI:** 10.3390/jcm9030871

**Published:** 2020-03-23

**Authors:** Muhammad Arsalan, Muhammad Owais, Tahir Mahmood, Jiho Choi, Kang Ryoung Park

**Affiliations:** Division of Electronics and Electrical Engineering, Dongguk University, 30 Pildong-ro 1-gil, Jung-gu, Seoul 04620, Korea; arsal@dongguk.edu (M.A.); malikowais266@gmail.com (M.O.); tahirmahmood.cs@gmail.com (T.M.); choijh1027@dongguk.edu (J.C.)

**Keywords:** cardiomegaly, cardiothoracic ratio, chest anatomy segmentation, X-Ray-Net

## Abstract

Automatic chest anatomy segmentation plays a key role in computer-aided disease diagnosis, such as for cardiomegaly, pleural effusion, emphysema, and pneumothorax. Among these diseases, cardiomegaly is considered a perilous disease, involving a high risk of sudden cardiac death. It can be diagnosed early by an expert medical practitioner using a chest X-Ray (CXR) analysis. The cardiothoracic ratio (CTR) and transverse cardiac diameter (TCD) are the clinical criteria used to estimate the heart size for diagnosing cardiomegaly. Manual estimation of CTR and other diseases is a time-consuming process and requires significant work by the medical expert. Cardiomegaly and related diseases can be automatically estimated by accurate anatomical semantic segmentation of CXRs using artificial intelligence. Automatic segmentation of the lungs and heart from the CXRs is considered an intensive task owing to inferior quality images and intensity variations using nonideal imaging conditions. Although there are a few deep learning-based techniques for chest anatomy segmentation, most of them only consider single class lung segmentation with deep complex architectures that require a lot of trainable parameters. To address these issues, this study presents two multiclass residual mesh-based CXR segmentation networks, X-RayNet-1 and X-RayNet-2, which are specifically designed to provide fine segmentation performance with a few trainable parameters compared to conventional deep learning schemes. The proposed methods utilize semantic segmentation to support the diagnostic procedure of related diseases. To evaluate X-RayNet-1 and X-RayNet-2, experiments were performed with a publicly available Japanese Society of Radiological Technology (JSRT) dataset for multiclass segmentation of the lungs, heart, and clavicle bones; two other publicly available datasets, Montgomery County (MC) and Shenzhen X-Ray sets (SC), were evaluated for lung segmentation. The experimental results showed that X-RayNet-1 achieved fine performance for all datasets and X-RayNet-2 achieved competitive performance with a 75% parameter reduction.

## 1. Introduction

The automatic segmentation of the chest anatomy is important for diagnosing pulmonary diseases, where the radiologist evaluates pulmonary discrepancies, such as nodules, lung deformation, and tissue mass disorders [1]. The chest X-Ray (CXR) is used world-wide for the chest analysis of several diseases, including pulmonary cancer, which is the leading cause of death [2]. The CXR is a common diagnostic tool used by doctors to detect various radiological signs. The lung shape features from the CXR can be used to diagnose pleural effusion, which is directly related to tuberculosis and congestive heart failure [3]. Considering the importance of chest anatomy, emphysema, which causes hyperinflation of alveoli, can be observed by the lung shape because a silhouette appearance of the lung field is created. Several studies have performed emphysema predictions using CXRs [4,5,6]. Cardiomegaly is a medical condition caused by hypertension, and it leads to an abnormal increase in the size of the heart. Cardiomegaly can be the result of artery disease, and it is the leading cause of sudden cardiac death [7]. Cardiomegaly can be assessed by the cardiothoracic ratio (CTR), which is measured manually by medical experts using the boundaries of the lungs and heart in CXRs [8]. Several studies have evaluated segmentation of the chest anatomy to estimate the CTR for cardiomegaly and related diseases [9,10,11,12,13]. To obtain advancement in diagnosis, automated systems are required to aid the medical specialist and overcome the diagnostic burden [2,3]. Most of the described diseases are related to the shape and size of the anatomical structures, which require accurate segmentation of the lung and heart boundaries. Lung segmentation benefits the diagnosis of diseases, such as cardiomegaly, emphysema, pleural effusion, etc., where heart segmentation can be used to determine the cardiothoracic ratio [1]. Automatic pulmonary disease detection using computer-aided diagnosis (CAD) is based on the correct segmentation of anatomical structures, such as the lungs, heart, and clavicle bones [2]. With the success of deep learning, artificially intelligent algorithms can help medical experts and ophthalmologists to detect and diagnose the disease and increase diagnostic throughput [14,15,16,17,18,19,20]. Semantic segmentation is a special branch of deep learning that involves pixel-wise classification of the image, which is important to accurately locate the infected areas for disease analysis [21,22]. Considering semantic segmentation of the CXRs, segmentation of the lungs, heart, and clavicle bones is challenging because of the low-quality images and low pixel variation. Previous studies evaluated these issues with preprocessing or deep networks that involve a lot of trainable parameters, creating a computationally expensive CAD solution [23,24]. This study focuses on the accuracy and computational cost for chest anatomy segmentation (lungs, heart, and clavicle bones) for diagnostic purposes. The accuracy of anatomical structure segmentation is enhanced by edge information empowerment by adding the spatial information from the preceding layers. The number of trainable parameters is reduced by reducing the trainable filters at the convolutional level in the encoder and decoder. The proposed solution is a learning-based method that is considered superior to conventional image processing methods that use specific thresholds and gray-levels in the image. This study is based on two separate semantic segmentation architectures, referred to as X-RayNet-1 and X-RayNet-2. X-RayNet-1 uses the residual mesh for better edge information flow to evenly segment the required anatomical structure with a small number of pixels (clavicle bones). X-RayNet-2 is visually the same as X-RayNet-1; however, the number of trainable parameters is substantially reduced on the layer level. X-RayNet-1 is based on 9.5 million trainable parameters, where X-RayNet-2 is based on 2.39 million parameters, exhibiting approximately a 75% parameter reduction. X-RayNet provides binary masks for the desired class, and the masks are used to compute the number of the pixel and the position to aid the medical diagnosis of various diseases.

Anatomical structure segmentation of the chest can be divided into two groups of conventional handcrafted features and deep feature-based methods. Starting from the baseline of handcrafted features-based methods that just consider the single class lung segmentation [2] using local features, researchers have mainly focussed on the general image processing-based methods for the chest anatomy segmentation, as presented in studies [25,26,27,28,29,30,31,32,33,34,35,36,37,38,39]. As this study is based on multiclass deep learning-based semantic segmentation, we mainly focus on learned feature-based literature.

The learned feature-based methods have been evaluated as an alternative to conventional image processing approaches. Dai et al. used the structural correcting adversarial network (SCAN) multiclass chest anatomy with critical learning of higher-order structures in limited data [40]. Dong et al. presented an adversarial network-based supervised learning method with domain adaption for estimation of a domain-independent output mask [41]. Tang et al. presented crisis-cross attention-based segmentation and X-Ray image synthesis, where the image-to-image translation module is responsible for data augmentation using multimodal unsupervised image-to-image translation (MUNIT) [42]. Souza et al. used a patch-based deep learning approach to lung region segmentation by using an AlexNet-similar structure. The classified pixels are plotted and reconstructed to obtain the fine boundaries [43]. Venkataramani et al. presented ContextNets with continuous domain adaption to train the network with a small number of images [44]. Novikov et al. utilized the famous U-Net-similar architecture and made a modification to specifically increase the segmentation performance for the heart and clavicle bone classes [1]. Solovyev et al. presented a novel method for the estimation of the CTR. They utilized the feature pyramid network (FPN) decoder with the change of batch normalization for instance normalization and to incorporate dropout in the network [12]. Oliveira et al. proposed transfer learning-based semantic segmentation for multiclass chest anatomy segmentation. They used pre-trained networks, such as fully convolutional networks (FCN), U-Net, and SegNet with transfer learning [45]. Islam et al. presented an efficient lung segmentation model. They also utilized the U-Net model to extract the lung region from the background. Several techniques were used to artificially increase the amount of data for training [46]. Wang et al. presented a multiclass CXR segmentation method with promising segmentation performance. They considered instance segmentation using mask-based region convolutional neural network (Mask-RCNN). They used ResNet50 and ResNet101 as the backbone network for Mask-RCNN [47]. Dong et al. presented deep learning for chest organ segmentation. They used a generative adversarial approach, in which the optimal discriminator design was proposed [48]. Jiang et al. presented deep convolution neural network-based segmentation using a small amount of data. They used a VGG16 network using prior weight initialization [49]. 

Table 1 lists the strengths and weaknesses of the existing methods in comparison to X-RayNet for chest anatomy segmentation.

This study evaluates two multiclass CXR segmentation networks (X-RayNet-1 and X-RayNet-2) to segment lung, heart, and clavicle bones to aid medical specialists in the diagnosis of cardiomegaly and other related diseases. Compared to existing works, this study is novel in the following four ways:

1. X-RayNet does not require preprocessing for multiclass semantic segmentation to detect the lungs, heart, and clavicle bones at the same time. X-RayNet considers the importance of computational cost; therefore, X-RayNet-2 reduces the trainable parameters by 75% with a competitive performance.
2. This study presents two separate identical semantic segmentation networks with a simple fully convolutional architecture.
3. X-RayNet utilizes a mesh of internal and external residual paths that transfers the enriched features from the preceding layers and at the end of the network. X-RayNet uses identity and nonidentity mappings for faster edge information transfer to ensure the residual mesh connects all the convolutional layers, including the first convolutional layer.
4. For a fair comparison with other research results, the trained X-RayNet models and algorithms are made publicly available in [50].

## 2. Materials and Methods

### 2.1. Overview of Proposed Architecture

**Figure 1:**
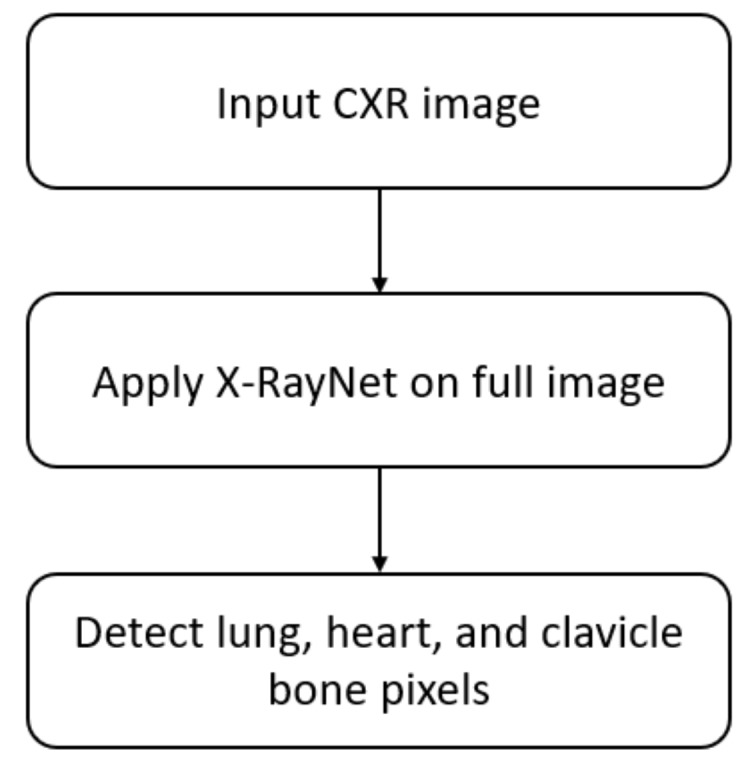
Flowchart of the proposed method.

Figure 1 shows an overview of the proposed method for chest anatomical structure segmentation. X-RayNet provides accurate multiclass segmentation of the lung, heart, and clavicle bones using pixel-wise classification for diagnostic purposes. The proposed method considers the importance of the enriched spatial edge information that resides in the initial layers of the network. The mesh-based residual paths provide this edge information to the next layers and outside the encoder. To utilize the benefits of identity and nonidentity mapping and to ensure the connectivity of each convolutional layer of the encoder with the residual mesh, identity mapping is used in the encoder, and nonidentity mapping is used in the decoder. The original image is directly provided to X-RayNet without conventional preprocessing, and it provides four output masks for each class of the lungs, heart, clavicle bones, and background. 

### 2.2. Chest Anatomy Segmentation Using X-RayNet

The classification by convolutional neural networks is the base of semantic segmentation, in which continuous convolutions are applied until the image is represented by the tiny features, and after classification, the image is upsampled again for the segmentation mask [51]. The continuous convolution also eliminates useful class spatial information during its process [51]. To preserve the important spatial information, the residual networks (ResNet) [52] have residual skip connections, which empower the features owing to ResNet’s attribute of superior performance of visual geometry group networks (VGG-Nets). The famous semantic segmentation network SegNet is based on VGG-Net, and it does not consider the residual connections; therefore, segmentation performance is lacking in the minor classes of the road scene, such as column/poles, sign/symbols, and bicyclists [53]. The spatial information loss is dealt with in X-RayNet by reducing convolutional blocks and using residual mesh.

**Figure 2:**
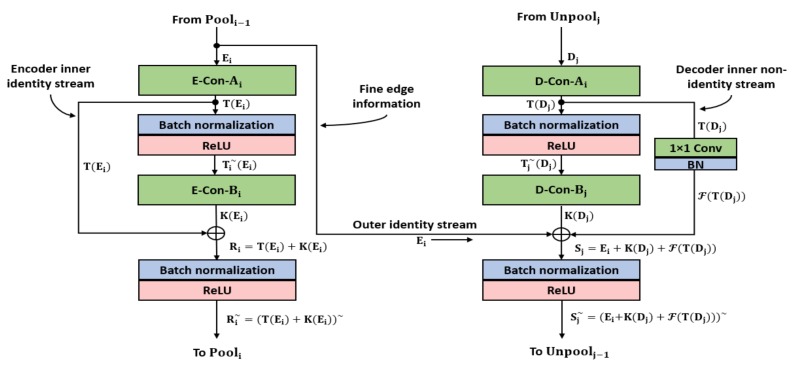
X-RayNet residual mesh schematic.

The CXR images do not have superior quality, and the edges of the chest organs are not clear. The segmentation in the CXR scenario is difficult in multiclass segmentation because of pixel differences. Unlike other traditional networks where the final feature map is small (7 × 7) [51], the X-RayNet maintains the final feature map at 21 × 21 for a 350 × 350 CXR image with a total of 17 layers overall. Table 2 lists the key differences of the proposed X-RayNet with deep networks, such as ResNet [52], SegNet [53], IrisDenseNet [54], fully residual encoder–decoder network (FRED-Net) [55], outer residual skip network (OR-Skip-Net) [56], Vess-Net [15], and U-Net [57], in different application domains. Considering the mesh residual structure of X-RayNet, Figure 2 shows the layer connectivity of the candidate encoder and decoder block with a feature empowerment scheme. According to Figure 2, each first encoder convolutional layer E-Con-$A_{i}$ receives the pooled feature $E_{i}$ from the pooling layer of the previous block ${\mathrm{Pool}}_{i-1}$ and provides the output $T(E_{i})$, which is later changed to $T_{i}^{\sim }(E_{i})$ after batch normalization, and ReLU, where this feature $T_{i}^{\sim }(E_{i})$ becomes $K(E_{i})$ after the second convolutional layer E-Con-$B_{i}$ operation. After the second convolution, the features $T(E_{i})$ and $K(E_{i})$ are added elementwise via an encoder inner identity stream (IIS) to create $R_{i}=$ $T(E_{i})$ + $K(E_{i}),$ which is later changed to $R_{i}^{\sim }$, given as the following equation.

(1) $$R_{i}^{\sim }={\left(T(E_{i})+K(E_{i})\right)}^{\sim }$$

Here, $R_{i}^{\sim }$ is the enhanced feature (available for the pooling layer of the current encoder block) that compensates for the loss of information created by E-Con-$A_{i}$; “+” represents the elementwise addition; “~” indicates the combined process of batch normalization and ReLU. The most important enriched feature (fine edge information) is $E_{i}$, which is a nonaltered feature directly from the pooling layer. Here, $E_{i}$ is directly fed to the corresponding decoder block by the outer identity stream (OIS). If $E_{i}$ is from the first block of the network, it contains real edge information of the feature. Similar to the encoder block, the first convolution of decoder block D-Con-$A_{i}$ receives the $D_{j}$ feature from the current unpooling layer ${\mathrm{Unpool}}_{j}$ and gives $T(D_{j})$ as an output feature, which later is changed to $T_{i}^{\sim }(D_{j})$ after batch normalization and ReLU operation. Feature $T_{i}^{\sim }(D_{j})$ becomes $K(D_{i})$ after the second convolutional layer D-Con-$B_{j}$ operation. After the second convolution in the decoder, features $F(T(D_{j}))$, $K(D_{j})$, and $E_{i}$ are added elementwise and are features from the decoder inner nonidentity stream (INIS) to create $S_{j}=$ $E_{i}+K(D_{j})+F(T(D_{j})),$ which is later changed to $S_{j}^{\sim }$, given by the following equation.

(2) $$S_{j}^{\sim }={\left(E_{i}+K(D_{j})+F(T(D_{j}))\right)}^{\sim }$$

Here, $S_{j}^{\sim }$ is the enhanced feature (available for the unpooling layer of the next decoder block), which compensates for the loss of information created by D-Con-$A_{j}$ and D-Con-$B_{j}$ and also empowers the feature with important edge information $E_{i}$ from the encoder; “+”represents elementwise addition; “~” indicates the combined process of batch normalization and ReLU. Here, $S_{j}^{\sim }$ is the feature that guarantees better segmentation edges and ensures the detection of the minor class, such as the clavicle bones, with accuracy.

**Figure 3:**
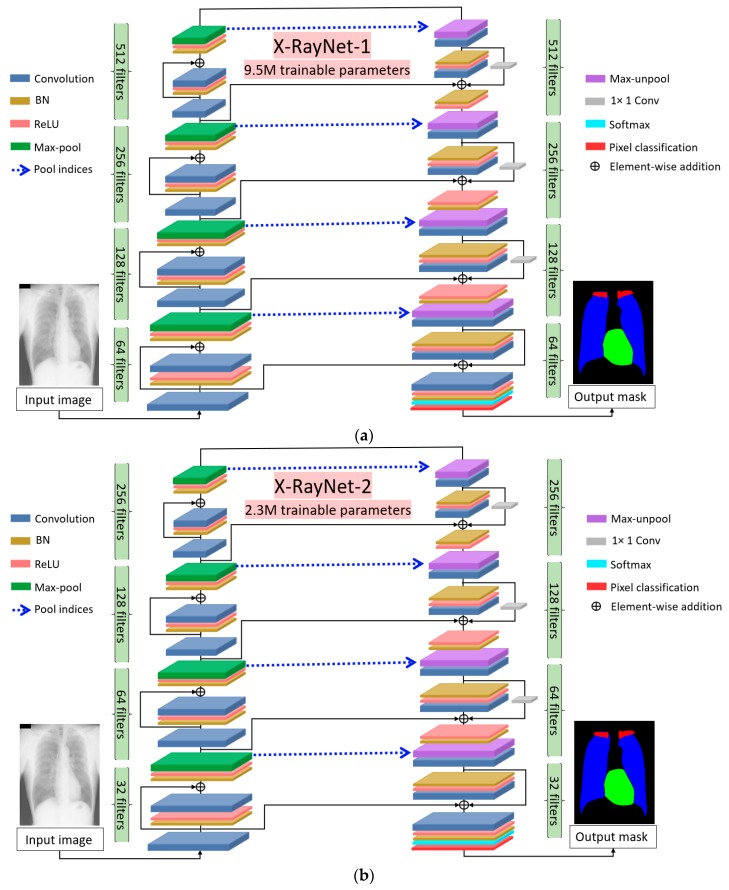
Proposed X-RayNet architecture for chest X-Ray (CXR) semantic segmentation: (**a**) X-RayNet-1 without filter reduction and (**b**) X-RayNet-2 with filter reduction.

Figure 3a,b shows the complete description of X-RayNet-1 and X-RayNet-2, respectively. Both networks are identical in terms of architecture; however, there is a large difference between the number of trainable parameters. To reduce the number of trainable parameters, there is no schematic difference created in the architecture; however, the number of filters in all the convolutional layers are halved.

#### 2.2.1. X-RayNet Encoder

Considering the encoder, there are a total of eight convolutional blocks for X-RayNet-1 and X-RayNet-2. In each encoder convolutional block, there are two convolutional layers that are connected to each other with the inner identity stream (IIS). ReLU is combined with batch normalization (BN) and exists after elementwise addition; thus, it exhibits postactivation. The encoder performs the continuous convolutional operation until the image is represented by the tiny feature for multiclass segmentation. The final feature map after the last max-pooling layer is 21 × 21; however, it is empowered to represent the fine features for all the classes. The X-RayNet-1 encoder starts the convolutional process with 64 filters and ends with 512 filters. X-RayNet-2 is a swift network that reduces the number of filters substantially. X-RayNet uses an RGB image of 350 × 350 pixels as an input, and at the end of the encoder, it provides an output of 21 × 21 pixels to the decoder for upsampling. 

The X-RayNet encoder structure is listed in the Appendix A section in Table A1, which shows that the residual mesh provides four residual skip connections by IIS and initiates four residual skip connections by OIS for the decoder. Table A1 also lists the feature map sizes and learnable parameters by each layer in the encoder.

#### 2.2.2. X-RayNet Decoder

Figure 3 shows the overall structure of X-RayNet. The X-RayNet decoder has two convolutions in each block; however, there are a few important changes. To provide connection to all convolutional layers in the decoder through the residual mesh, most of the internal residual connections in the decoder are based on nonidentity mapping (except the last convolutional block in the decoder). Moreover, one additional convolutional layer at the end of the network is added for the class masks. The X-RayNet decoder receives the 27 × 27-pixel feature from the last pooling layer of the encoder and provides the output mask of 350 × 350, similar to the size of input image. Considering the decoder of X-RayNet-2, the architectural scheme is similar to X-RayNet. As explained earlier, the number of filters in X-RayNet is reduced by half to reduce the number of trainable parameters. for practical scenarios. After the last convolutional block, there is an output block that contains one convolution layer for the class masks and a combination of softmax and pixel classification layer. The purpose of the pixel classification layer is to assign a pixel label for each class according to the loss. Table A2 lists the layer structure and feature map sizes for the X-RayNet decoder.

## 3. Results

### 3.1. Experimental Data and Environment

**Figure 4:**
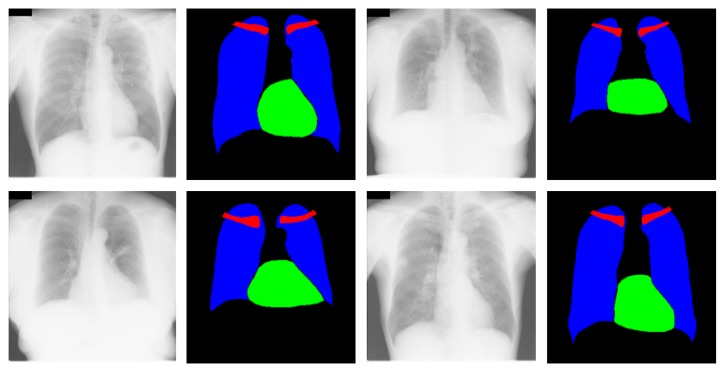
Sample CXR images and ground truths for the Japanese Society of Radiological Technology (JSRT) dataset.

This research focused on multiclass chest anatomy segmentation. Therefore, the segmentation performance of the proposed X-RayNet was tested on a publicly available multiclass dataset released by the Japanese Society of Radiological Technology (JSRT) [58]. The JSRT dataset consists of a total of 247 CXRs for research purposes. The multiclass pixel-level annotation for the lungs, heart, and clavicle bones was provided by Van Ginneken et al. [59], and these annotations are used for training and testing of the proposed network. Specifically, two observers familiar with medical image analysis manually segmented the chest objects with instructions from an experienced radiologist; both observers reviewed the results repeatedly until the radiologist was convinced that the segmentation was reliable. The original size of the images is 2048 × 2048 pixels with 0.175 mm of pixel space. Out of 247 images, 154 images contain nodules, where the remaining 94 images do not contain lung nodules. The JSRT is available in two folds of 124 and 123 images, respectively. In this study, one fold was used for training and the other fold was used for testing based on the two-fold cross-validation criteria used in [47]. Then, the final accuracy was calculated by averaging the accuracies of both folds. Figure 4 shows example CXRs from the JSRT dataset with corresponding multiclass ground truth for the lungs, heart, and clavicle bones. The blue, green, and red pixels show the lung, heart, and clavicle bone pixels, respectively. 

To reduce the training time and graphic processing unit (GPU) memory usage, the images and labels of JSRT were evenly resized to 350 × 350 pixels. The X-RayNet is a semantic segmentation network that performs pixel-wise classification. This pixel-wise classification requires a large amount of training data, which is artificially created by data augmentation explained in Section 3.2.

X-RayNet was trained and tested on a desktop with Intel^®^ Core™ i7-3770K CPU with the clock speed of 3.50 GHz (4 cores). The system RAM was 28 GB with NVIDIA GeForce GTX Titan X GPU (3072 Cuda cores with a graphics memory of 12 GB) [60]. In our experiments, X-RayNet was designed and trained from scratch using MATLAB 2019a [61](without fine-tuning of a pretrained model, such as ResNet, GoogleNet, Inception, or DenseNet.) 

### 3.2. Data Augmentation

**Figure 5:**
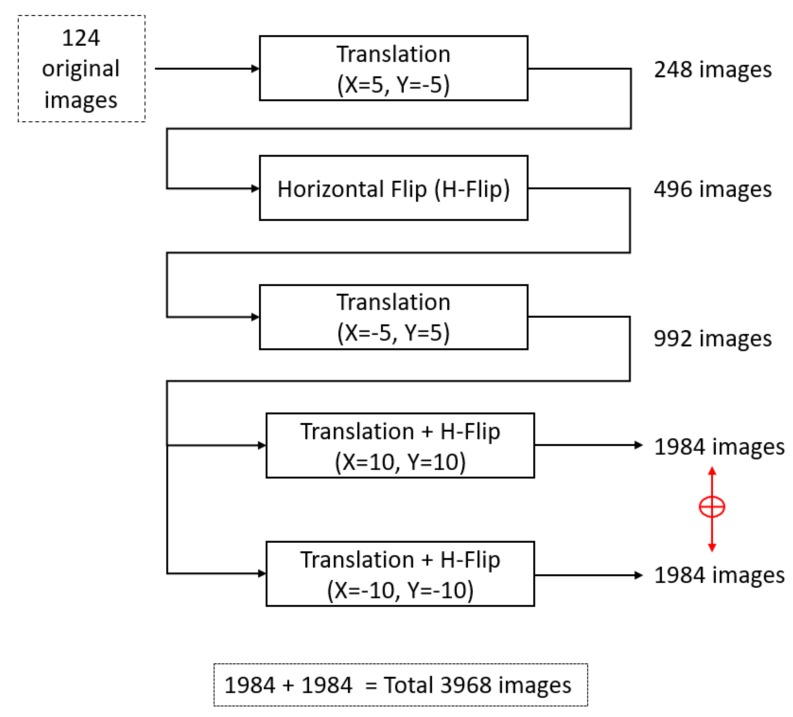
Data augmentation strategy used to artificially increase the training data; H-Flip represents the horizontal flip.

To train a semantic segmentation network sufficiently, a large amount of labeled data is required, which is difficult to arrange in all scenarios. Considering the medical domain, the datasets are difficult to label because expert knowledge is required. Thus, different data augmentation schemes are used to artificially increase the amount of data or to create a variety for network learning. To train X-RayNet and X-RayNet-2 with a variety of images and guarantee successful learning, artificial images were created with several image transformations, such as cropping, resizing, and horizontal flipping with interpolation. The basic schematic of the proposed data augmentation is shown in Figure 5. 

Using a total of 124 images, the first step X-Y translation with (X = 5, Y = −5) was applied without flipping, resulting in 248 images. In the second step, the 248 images from the previous step were flipped horizontally to create a total of 496 images. In the third step, the 496 images from the previous step were X-Y translated (X = −5, Y = 5) with a horizontal flip, resulting in a total of 992 images. In the fourth step, two different transformations of (X = 10, Y = 10) with the horizontal flip and (X = −10, Y = −10) with the horizontal flip were applied to the 992 images from the previous stage, resulting in 1984 and 1984 images, respectively. Therefore, with the combination of transformational images from step four, a total of 3968 (1984 + 1984) images were obtained, which were used for training purposes (as shown in Figure 5).

### 3.3. X-RayNet Training

**Figure 6:**
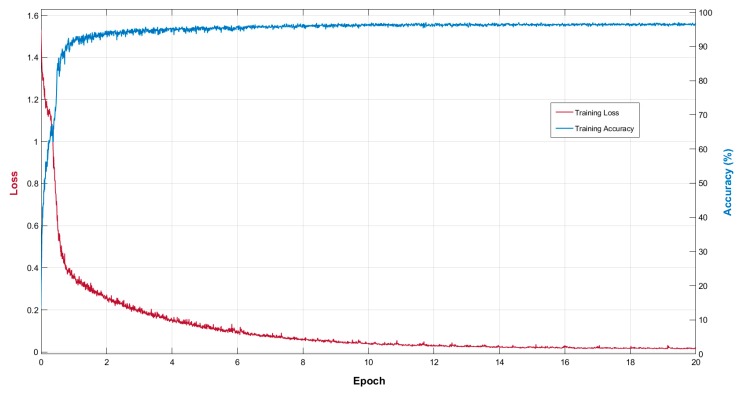
Training loss and accuracy curve (per epoch) for X-RayNet.

X-RayNet is based on a residual mesh, which provides the network with several residual paths for the encoder-decoder internal and external connectivity and helps the network to quickly converge. The spatial edge information through the residual mesh provides fine edge segmentation to avoid the preprocessing overhead. The training of X-RayNet was performed from scratch without prior weight transfer or initialization. X-RayNet is our designed network; therefore, to train X-RayNet, finetuning was not used from the conventional models. The Adam optimizer is a well-known version of stochastic gradient descent (SGD), and it provides efficient performance for diagonal scaling of the gradient, suitable for larger data, and even good for moving object classification problems [62]. Because of the benefits of Adam, it was adopted as an optimizer to train X-RayNet. Considering the other training parameters, the initial learning rate was 0.0003, which was maintained during the training of 20 epochs (34,440 iterations). The X-RayNet design has a low memory requirement; therefore, a minibatch size of 17 images was used for the training. The global L2 normalization with an epsilon of 0.000001 was used as the gradient threshold method, where the gradient threshold of six was maintained during the training. The CXR images are multiclass with a different number of pixels per class; thus, the cross-entropy loss with median frequency balancing was used to quickly train the network. A similar scheme of cross-entropy in combination with frequency balancing was utilized in [53,54,55,56]. Figure 6 shows the training loss and accuracy curves for the proposed X-RayNet. The x-axis represents the number of epochs. The training loss is presented on the left y-axis (red color), and the training accuracy is presented on the right y-axis (blue color). The loss and accuracy are shown on the basis of the minibatch of 17 images per epoch. The X-RayNet training of 20 epochs with a learning rate of 0.0001 and minibatch size of 17 images achieved the training accuracy of approximately 97% with a training loss of approximately 0.01. As described in Section 1, the X-RayNet trained models will be made publicly available to allow comparison with other studies via [50].

### 3.4. Testing of the Proposed Method

#### 3.4.1. X-RayNet Testing for Chest Anatomy Segmentation 

As stated in Section 1, X-RayNet does not require preprocessing of the image using conventional image processing schemes for training and testing. The original image with a size of 350 × 350 was directly provided to X-RayNet, where the network performed the continuous convolution process to classify the object available classes in a feed-forward fashion. The continuous process degrades the image on each step; however, the residual mesh, which consists of an OIS, IIS, and INIS, ensures compensation for the lost feature with residual paths, as shown in Figure 3 and listed in Table A1 and Table A2. X-RayNet enhances the feature from the preceding layers using 12 different internal and external residual skip paths. At the X-RayNet output, the convolution layer was used with four filters (MConv, as listed in Table A2), in which each channel represents the separate classes of the lung, heart, clavicle bones, and background. Thus, the output of X-RayNet is the four masks for each individual class output. To evaluate the segmentation performance by the proposed X-RayNet, the accuracy (Acc); mean intersection of union (mIOU), which is also referred as the Jaccard index (J); and dice coefficient (D) were measured, which were similarly utilized by [1,12,47] to evaluate and compare the JSRT dataset with other methods. The formulas for J and D are given by Equations (3)–(5).

(3) $$\mathrm{Acc}=\frac{TP+TN}{TP+FP+FN+TN}$$

(4) $$J=\frac{TP}{TP+FP+FN}$$

(5) $$D=\frac{2TP}{2TP+FP+FN}$$

Here, *TP*, *FP*, and *FN* are the numbers of true positives, false positives, and false negatives, respectively. Considering the example of one class of lungs, the *TP* pixels are the pixels that are predicted as lung pixels and listed as lung pixels in the ground truth. The *FP* pixels are the pixels that are predicted as lung pixels and listed as a nonlung pixels in the ground truth. The *FN* pixels are the pixels that are predicted as nonlung pixels by our network but listed as lung pixels in the ground truth. 

#### 3.4.2. Chest Organ Segmentation Results by X-RayNet

**Figure 7:**
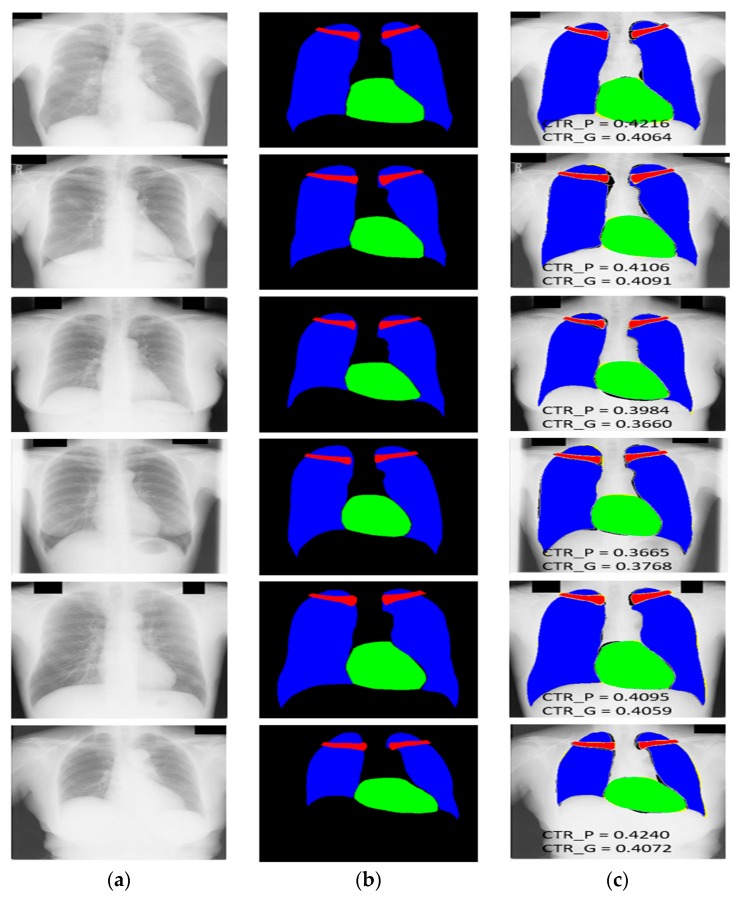
Examples of chest anatomical structure segmentation by X-RayNet for the JSRT dataset: (**a**) original CXR image; (**b**) ground-truth mask; (**c**) predicted mask result by X-RayNet; false positives (*FP*) (shown in black for each class), false negatives (*FN*) (shown in yellow for each class), and true positives (*TP*) (shown in blue, green, and red for the lung, heart, and clavicle bone classes, respectively). CTR_P and CTR_G represent the CTR predicted by the proposed method and CTR by ground-truth mask.

Figure 7 shows the multiclass segmentation results of the CXR images by X-RayNet with the JSRT dataset and CTR predicted (CTR_P) with the proposed method, and the CTR with ground truth (CTR_G) mask provided by [59] in the supervision of expert radiologist using the same criteria. Figure 7 shows the convention of *FP* (shown in black for each class), *FN* (shown in yellow for each class), and *TP* (shown in blue, green, and red for the lung, heart, and clavicle bone classes, respectively). Considering the bad segmentation cases, there is no considerable segmentation error for the test images using our method. 

#### 3.4.3. Comparison of X-RayNet with Other Methods

In this section, the segmentation performance comparisons between X-RayNet and other methods are compared based on the performance measure of J and D described in Section 3.4.1. Table 3 lists the segmentation performance comparisons of the existing method with those obtained by X-RayNet for the JSRT dataset. The results prove the superior performance of X-RayNet for chest anatomy segmentation compared to current studies, based on the values of J and D. The comparison in Table 3 lists the local feature-based methods and learned feature-based methods separately.

#### 3.4.4. Lung Segmentation with Other Open Datasets Using X-RayNet

**Figure 8:**
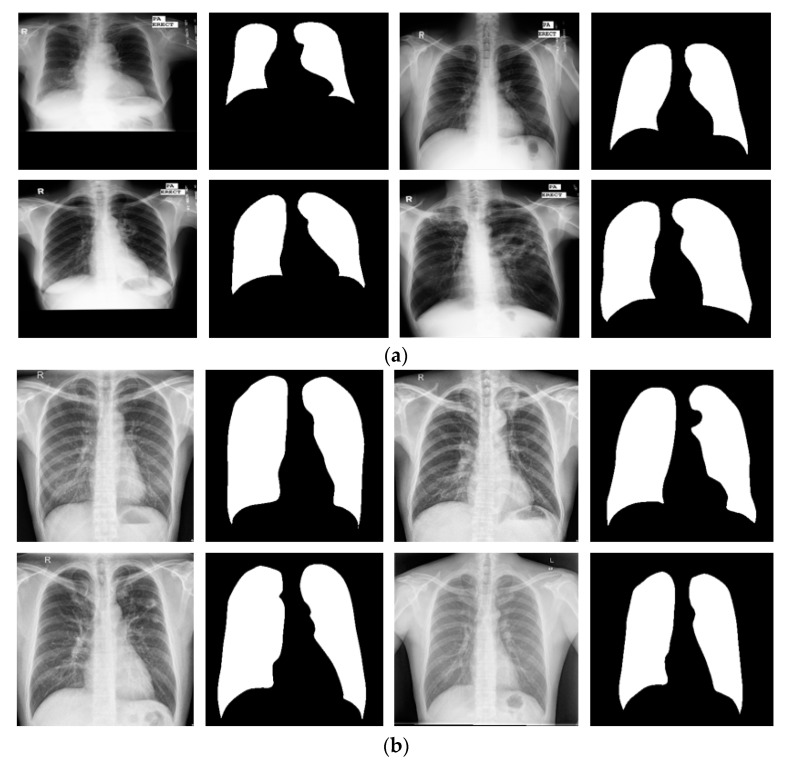
Examples of X-Ray images from the (**a**) Montgomery County chest X-Ray set (MC) and (**b**) Shenzhen chest X-Ray set (SC) datasets with corresponding ground truths.

To evaluate the segmentation performance with X-RayNet in different image acquisition conditions, this study included experiments with two additional publicly available datasets of lung segmentation: the Montgomery County chest X-Ray set (MC) [63] and Shenzhen chest X-ray set (SC) [63]. MC consists of l38 frontal chest X-Ray images from the Montgomery County tuberculosis program run by the department of health and human services of Montgomery County, Maryland, USA. The MC dataset consists of 80 normal and 58 tuberculosis cases, where the X-Ray images were obtained using a Eureka stationary X-Ray machine. The images are provided in PNG format along with the lung contour binary mask as ground truth (as shown in Figure 8a). The SC dataset is from Shenzhen No. 3 People’s Hospital of Guangdong Medical College, Shenzhen, China. The SC dataset consists of 662 frontal chest X-Ray images with 326 normal and 336 tuberculosis manifestation cases, where the images were obtained using a Philips DR Digital Diagnostic System. In our experiments with MC, we followed the same criteria as [43]. For the 138 images, 80 images were used for training, 20 images for validation, and 38 images for testing purposes. Considering the SC dataset with the provided 662 images, the lung mask of 566 images was provided with the dataset. From the 566 images, 50% of the images (283) were used for training, and the remaining images (283) were used for testing purposes with a two-fold cross-validation. Example images of the MC and SC dataset with the ground truth image are shown in Figure 8a,b), respectively. To train X-RayNet with MC and SC, similar data augmentation was used as described in Section 3.2. The ground-truth mask for MC and SC are provided for only the lungs. 

**Figure 9:**
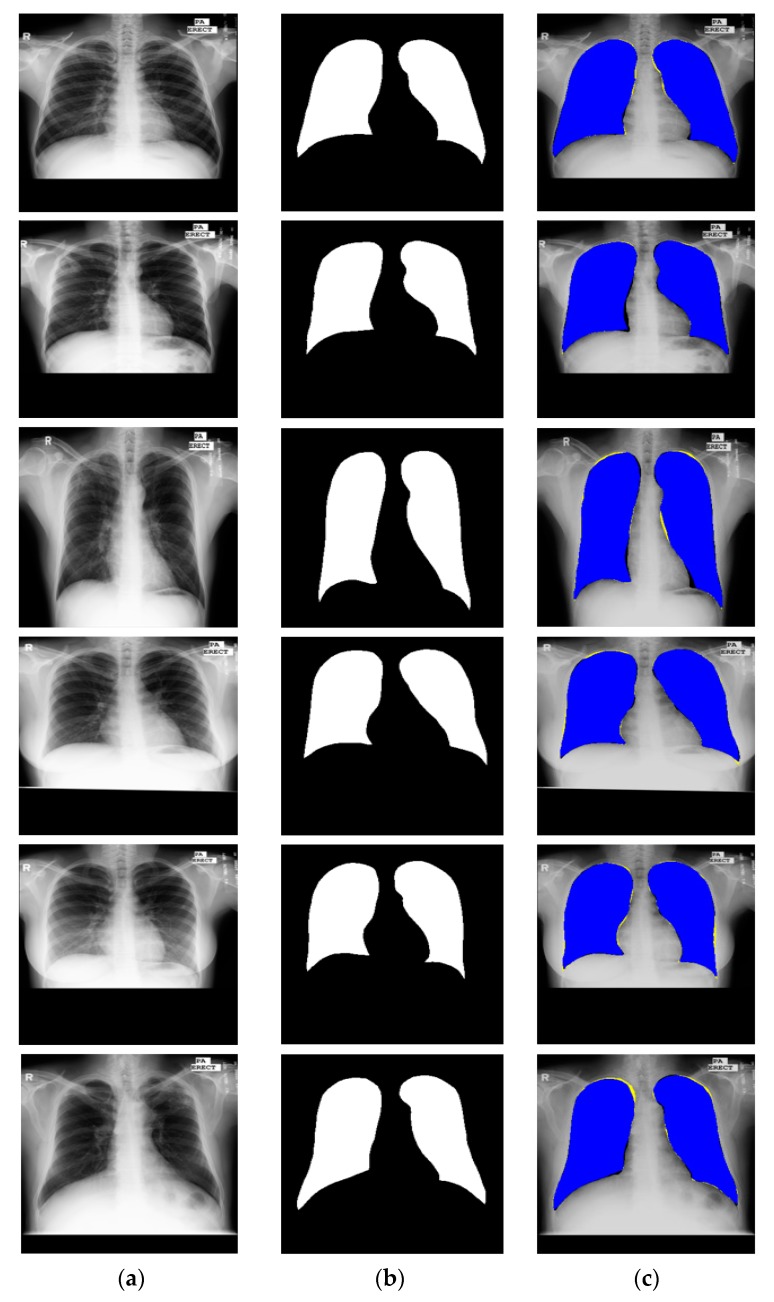
Examples of lung segmentation by X-RayNet for the MC dataset: (**a**) original image; (**b**) ground-truth mask; (**c**) segmented image by X-RayNet (*TP* is presented in blue, *FP* in black, and *FN* in yellow).

**Figure 10:**
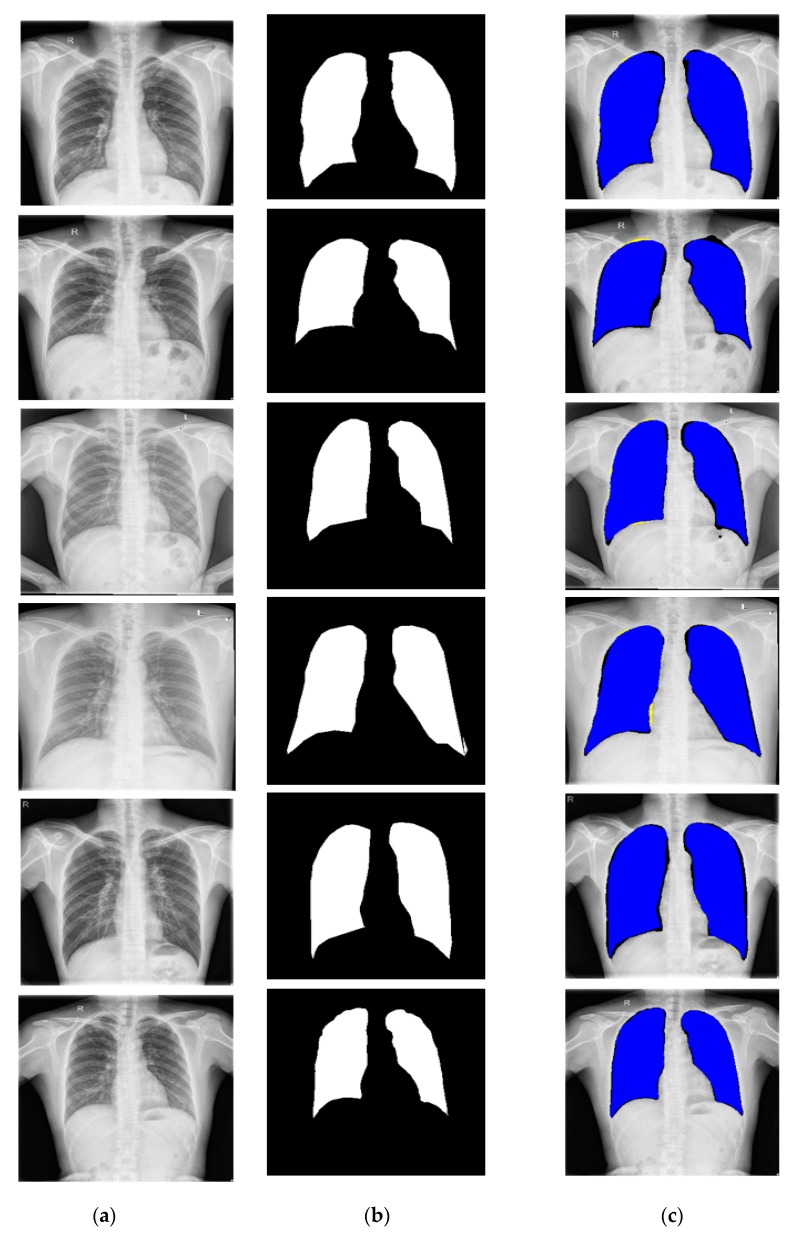
Examples of lung segmentation by X-RayNet for the SC dataset: (**a**) original image; (**b**) ground-truth mask; (**c**) segmented image by X-RayNet (*TP* is presented in blue, *FP* in black, and *FN* in yellow).

Figure 9 and Figure 10 show the segmentation results by X-RayNet for the MC and SC datasets with the areas of *TP*, *FN*, and *FP*. Considering the bad segmentation performance case, X-RayNet is powered by the residual mesh; therefore, there is no significant segmentation error or nonsegmentation case for the MC and SC datasets. Table 4 and Table 5 list the experimental result comparison for X-RayNet-1 and X-Ray-Net-2 with existing studies based on the MC and SC datasets. The experimental results validate the fine performance of X-RayNet for lung segmentation, which will be used for diagnostic purposes.

Separate training and testing were performed in the experiment to provide a fair comparison with existing studies (based on the same experimental protocol), as listed in Table 3, Table 4 and Table 5. From Table 3, Table 4 and Table 5, our proposed X-RayNet-1 and X-RayNet-2 outperformed the state-of-the-art methods for chest anatomy segmentation for all three datasets. To test the portability of the proposed X-RayNet, two additional experiments were performed. In the first, X-RayNet was trained on MC and tested on SC. In the second experiment, X-RayNet was trained on SC and tested on MC. For these two cross-dataset experiments, the network was trained individually without any heuristic of the testing data. Table 6 lists the portability of our method. The performance of X-RayNet is sufficiently good for the training and testing of different datasets. The performance numbers in Table 6 show that the degradation in performance is small and better than the numerous state-of-the-art methods for lung segmentation. 

## 4. Discussion

**Figure 11:**
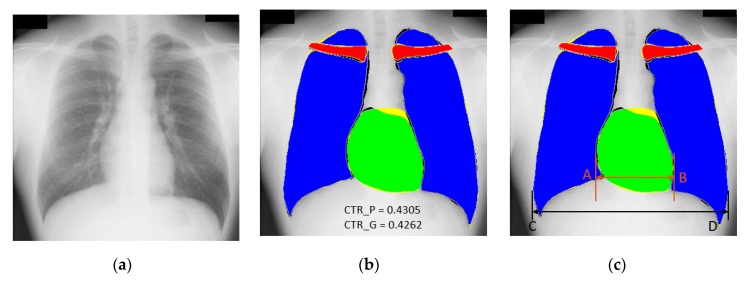
Sample image of chest anatomy segmentation for pixel count: (**a**) original image, (**b**) predicted mask by X-RayNet (*FP* (shown in black for each class), *FN* (shown in yellow for each class), and *TP* (shown in blue, green, and red for the lung, heart, and clavicle bone classes, respectively)), and (**c**) procedure for calculating CTR, CTR_P, and CTR_G represent the CTR predicted by the proposed method and that predicted by the ground-truth mask.

As described in Section 1, chest radiography is one of the most common diagnostic schemes to analyze multiple cardiothoracic and pulmonary diseases. The automatic detection of disease and CAD is an important aspect to reduce the workload of the medical practitioner. CTR can be used as the diagnostic tool for related diseases such as cardiomegaly which is a medical condition in which the heart size is increased, and this enlargement of the heart is estimated by CTR. The computation of CTR is normally performed by the medical practitioner manually using a visual analysis of the CXRs. This process of CTR computation can be automated by our proposed semantic segmentation network (X-RayNet), which segments the lungs and heart boundary accurately. In X-Ray images, the heart boundary is crucial as there is a only small change in pixel values as shown in Figure 11a. The CTR calculation by our method depends upon the clear boundary segmentation of the heart and lungs. The exact boundary segmentation is required even with minor changes of pixel values, which is effectively helped with feature empowerment. Figure 11a–c show an example image, a segmentation result by X-RayNet, and the CRT computation schematic for the JSRT dataset. Based on [13,41], CTR estimation was performed with the ratio of distance A⃡B and C⃡D. Here, A⃡B is the distance between two extreme points A and B for the heart, and C⃡D is the distance between two extreme outer points C and D for both lungs, as shown in Figure 11c and Equation (6).

(6) $$\mathrm{CTR}=\frac{A⃡B}{C⃡D}$$

Considering the specific example provided in Figure 11, the distance A⃡B by our method is 130 pixels, where the distance C⃡D is 302 pixels. In this example case, the predicted CTR (CTR_P) calculated using Equation (6) is 0.4305 where the CTR ground truth (CTR_G) calculated by the ground-truth mask provided by [59] under supervision by an expert radiologist using Equation (6) is 0.4262. According to [41], the CTR threshold can vary for different age groups explained by [13], and the determination of cardiomegaly through the CTR value can be automatically made by our method. Brakohiapa et al. [13] explained that the CTR is one of the main parameters that can be used for the detection of heart failure and cardiomegaly. The computational criteria related to age and gender for the CTR is effectively discussed for cardiomegaly in [13].

The proposed X-RayNet is a learning-based method in which the network learns the weights from training data. This trained knowledge is then utilized to predict the pixel-wise classes in the testing X-Ray image. The learning method-based predictions are subject to training knowledge; therefore, the misclassification of pixels can create a prediction error for the CTR computations. In addition, our method shows that *FN* cases occur more frequently than *FP* cases especially in the upper areas of heart, as shown in Figure 11b, because of the indistinctive boundary of the heart. Nevertheless, these errors do not affect the correct calculation of CTR because the CTR is calculated based on the horizontal distances of heart and lungs as shown in Equation (6). The proposed method can aid the medical practitioner for the diagnosis with CTR and the analysis of the segmented chest anatomy as a second opinion system.

## 5. Conclusions

This study proposed a residual mesh-based semantic segmentation network (X-RayNet) to segment the chest anatomical structures (lungs, heart, and clavicle bones) for diagnostic purposes. The method provides fine segmentation performance in nonideal scenarios and multiclass fashions. The innovative residual mesh design preserves the spatial edge information, which is provided throughout the network. The segmentation of the heart is crucial because the pixel value is low, and the edges mix with the lung borders. X-RayNet maintains feature empowerment to accurately segment the heart in inferior quality X-Ray images caused by the indistinctive boundaries of the heart. The accuracy of segmentation (for the heart and lungs area) is directly related to the correct computation of the CTR. The conventional convolutional neural networks reduce the feature map size to classify the classes. In this scenario, the minor information (clavicle bones and small-sized heart) vanishes owing to excessive use of the max-pooling layers. X-RayNet is designed to not reduce the feature map size for classification purposes. It uses a smaller number of pooling layers and maintains a sufficiently large final feature map to retain the minor class information. The performance of the minor class segmentation is listed in Table 3 for the clavicle and heart classes. The direct outer residual connection by the residual mesh causes direct information transfer, which enables X-RayNet to converge faster in merely 20 epoch (3440) iterations. X-RayNet-2 is a standalone complete variant of X-RayNet, in which the number of filters is optimized to reduce the total number of trainable parameters. Following similar residual mesh-based connectivity, X-RayNet-2 has sufficiently good segmentation performance with 75% reduction (compared to X-RayNet-1) of the trainable parameters, as shown in Figure 3a,b. The automated design of our proposed method can accurately determine the boundaries of the lungs and heart to reliably measure the CTR. The correctness of segmentation is directly proportional to the correctness of the CTR value. The CTR is considered a special parameter used to diagnose multiple cardiac and pulmonary diseases. 

X-RayNet creatively segments the boundaries with intersections and feature empowerment. In the future, we will create a similar low-cost network with separable convolutions to ensure sufficiently good segmentation performance with a low number of trainable parameters. In addition, X-RayNet can be used for other medical applications, such as semantic segmentation of brain tumors, melanoma, and orthopedic tasks.

## Figures and Tables

**Table 1:** Comparison of previous methods and X-RayNet for chest anatomy segmentation.

Type	Methods	Strength	Weakness
Using handcrafted local features *	Lung segmentation using Hull-CPLM [2]	Selects the ROI for lung detection	Preprocessing is required
	Nongrid registration lung segmentation [25]	Sift-flow modeling for registration provides an advantage	Boundary refinement is required
	Probabilistic lung shape model [26,32,35]	Probabilistic shape model mask helps in shape segmentation	Single threshold creates the segmentation error
	Otsu thresholding [27]	Excludes the noise area for lung nodule segmentation	Gamma correction is required
	Fuzzy c-means clustering [28,30,37]	Better performance compared to K-means	The lower value of β requires more iterations
	Active contour and morphology [29,39]	Active contour can estimate the real lung boundary	The iterative method takes many iterations
	Salient point-based lung segmentation [31,33]	Interpolation of salient points approximate lung boundary well	Results are affected by overlapped regions
	Harris corner detector [34,36]	Convolutional mask refines the contour	Edge detection is affected by noise
	Region growing [38]	Region growing methods are good towards the real boundary	ROI is required
**Using features based on machine learning or deep learning**	Structural correcting adversarial network [40,49]	Adversarial training is good for a small number of training images	Critic network requires fully connected layer and consumes a lot of parameters
	Domain adaptation [41,44]	Domain adaption is good to enhance segmentation performance	FCN-based segmentation consumes many parameters
	Lung segmentation by criss-cross attention [42]	Image-to-image translation is used for augmentation	Three separate deep models of ResNet101, UNet, and MUNIT are used
	Similar structure as AlexNet [43]	Semantic segmentation is close to real boundary	Patch-based deep learning scheme is computationally expensive
	FCN, U-Net, and SegNet for CXR segmentation [45]	Semantic segmentation provides good results for multiclass segmentation	FCN consumes many trainable parameters owing to fully connected layer
	U-Net [46]	U-Net is popular for medical image segmentation	Preprocessing is required
	Mask-RCNN [47]	Multiclass efficient segmentation is performed	Region proposals are also required with pixel-wise annotation
	ResNet [49]	Dropping 5^th^ convolutional block from VGG-16 reduces the number of parameters	Clavicle bone segmentation is not considered
	X-RayNet (proposed)	12 residual mesh streams enhance features to provide good segmentation performance	Data augmentation is required to artificially increase the amount of data

* Handcrafted local featuresare with conventional image processing schemes.

**Table 2:** Key architectural differences between X-RayNet and previous approaches.

Method	Other Architectures	X-RayNet
ResNet [52]	Only adjacent convolutional layers have residual skip paths	Both adjacent and nonadjacent layers have residual skip connections. There are paths between the encoder and decoder.
	1 × 1 convolution is employed as bottleneck layer in all ResNet variants	1 × 1 convolution is used to connect three blocks of the decoder based on nonidentity mapping
	Max-pooling layers are without indices information	Max-pool to max-unpool indices information is shared between the corresponding encoder and decoder block
	All variants use fully connected layers for classification purposes	The fully connected layers are not used to make the network a fully convolutional network (FCN) for semantic segmentation
	Average pooling is employed at the end of the network	Max-pooling layers and max-unpooling layers are used in each encoder and decoder block
IrisDenseNet [54]	Encoder and decoder consist of 13 convolutional layers each, resulting in a total of 26 convolutional layers	Encoder and decoder consist of eight and nine (3 × 3) convolutional layers, respectively
	Uses dense connectivity in encoder with depth-wise concatenation	Residual connectivity between encoder and decoder by elementwise addition
	First two blocks have two convolutional layers and the rest of the blocks have three convolutional layers in the encoder and decoder	Two convolutional layers in each encoder and decoder convolutional block, where one convolutional layer is at the end of the network to produce respective class masks
	The decoder is the same as the VGG-16 network without feature reuse by dense connectivity	Both encoder and decoder use the residual mesh connectivity for feature reuse
FRED-Net [55]	Only uses residual skip connections between adjacent convolutional layers of same block	Uses residual skip connections for adjacent convolutional layers and between encoder and decoder externally
	There is no skip connection between encoder and decoder	Inner and outer residual connections for spatial information flow
	The overall network has six skip paths	The overall network has 12 residual skip paths that create the residual mesh
	Overall network is based on nonidentity mapping	Among the 12 residual paths that create a residual mesh, nine are with identity mapping and three are with nonidentity mapping
	The ReLU is used after the elementwise addition that represents the postactivation only	The network is based on pre- and post-activation
SegNet [53]	26 convolutional layers	17 convolutional layers
	No residual connectivity that causes vanishing gradient problem	Vanishing gradient problem is handled by residual mesh
	Each block has a different number of convolutional layers	All the blocks have the same two convolutional layers
	512-depth block used twice to increase the depth of the network	Used 512-depth block once for X-RayNet-1 and 512-depth block is not used in X-RayNet-2
OR-Skip-Net [56]	There is no internal connectivity between the convolutional layers in the encoder and decoder	Both encoder and decoder convolutional layers are connected with residual mesh for feature empowerment
	The outer skip connections are with nonidentity mapping	The encoder-to-decoder connections are with identity mapping
	Only pre-activation is used as ReLU exists before elementwise addition	The network is based on pre- and post-activation
	Four residual connections are used	12 residual skip connections are used
Vess-Net [15]	16 convolutional layers are used	16 convolutions are used with an extra convolution in the decoder for fine edge connectivity
	The first convolutional layer has no internal or external residual connection	The features from the first convolutional layer are important for edge information for the minor class, like the clavicle bones; therefore, it is internally and externally connected
	All the convolutional layers are internally connected with each other inside the encoder and decoder with nonidentity mapping	Most of the internal layers of the encoder and decoder are connected using identity mapping
	10 residual paths	12 residual paths
U-Net [57]	23 convolutional layers are used	17 convolution layers are used
	Up convolutions are used in the expansive part for upsampling	The unpool layer in combination with normal convolution is used for upsampling
	1 × 1 convolution is used at the end of the network	1 × 1 convolution is only used in the decoder internal residual connections
	Feature concatenation is utilized for empowerment	Feature elementwise addition is utilized for feature empowerment
	Cropping is required owing to border pixel loss during convolution	The feature map size is controlled by indices information transfer between pooling and unpooling layers

**Table 3:** Accuracies of X-RayNet and existing methods for the JSRT dataset (unit: %).

Type	Method	Lungs			Heart			Clavicle Bones		
		Acc	J	D	Acc	J	D	Acc	J	D
**Local feature-based methods**	Peng et al. [22]	97.0	93.6	96.7	-	-	-	-	-	-
	Candemir et al. [25]	-	95.4	96.7	-	-	-	-	-	-
	Jangam et al. [28]	-	95.6	97.4	-	-	-	-	-	-
	Wan Ahmed et al. [30]	95.77	-	-	-	-	-	-	-	-
	Vital et al. [29]	-	-	95.9	-	-	-	-	-	-
	Iakovidis et al. [31]	-	-	91.66	-	-	-	-	-	-
	Chondro et al. [38]	-	96.3	-	-	-	-	-	-	-
	Hybrid voting [59]	-	94.9	-	-	86.0	-	-	73.6	-
	PC post-processed [59]	-	94.5	-	-	82.4	-	-	61.5	-
	Human Observer [59]	-	94.6	-	-	87.8	-	-	89.6	-
	PC [59]	-	93.8	-	-	81.1	-	-	61.8	-
	Hybrid ASM/PC [59]	-	93.4	-	-	83.6	-	-	66.3	-
	Hybrid AAM/PC [59]	-	93.3	-	-	82.7	-	-	61.3	-
	ASM tuned [59]	-	92.7	-	-	81.4	-	-	73.4	-
	AAM whiskers BFGS [59]	-	92.2	-	-	83.4	-	-	64.2	-
	ASM default [59]	-	90.3	-	-	79.3	-	-	69.0	-
	AAM whiskers [59]	-	91.3	-	-	81.3	-	-	62.5	-
	AAM default [59]	-	84.7	-	-	77.5	-	-	50.5	-
	Mean shape [59]	-	71.3	-	-	64.3	-	-	30.3	-
	Dawoud [35]	-	94.0	-	-	-	-	-	-	-
	Coppini et al. [4]	-	92.7	95.5	-	-	-	-	-	-
Deep feature-based methods	Dai et al. FCN [40]	-	92.9	96.3	-	86.5	92.7	-	-	-
	Dong et al. [41]		95.5	-			90.2			
	Mittal et al. [24]	98.73	95.10	-	-	-	-	-	-	-
	Oliveira et al. FCN [45]		95.05	97.45		89.25	94.24		75.52	85.90
	Oliveira et al. U-Net [45]		96.02	97.96		89.21	94.16		86.54	92.58
	Oliveira et al. SegNet [45]		95.54	97.71		89.64	94.44		87.30	93.08
	Novikov et al. InvertedNet [1]		94.9	97.4		88.8	94.1		83.3	91.0
	ContextNet-1 [44]		95.8	-	-	-	-	-	-	-
	ContextNet-2 [44]	-	96.5	-			-	-	-	-
	ResNet50 (512, C = 4) ~* [47]		93.9	96.8		88.3	93.7		79.4	88.3
	ResNet50 (512, C = 4) * [47]		95.3	97.6		89.4	94.3		84.9	91.8
	ResNet50 (512, C = 6) * [47]		94.5	97.2		89.3	94.3		84.3	91.5
	ResNet50 (512, C = 8) * [47]		94.9	97.4		89.7	94.5		84.7	91.6
	ResNet101 (512, C = 4) * [47]		95.3	97.6		90.4	94.9		85.2	92.0
	ResNet50 (256, C = 4) * [47]		95.0	97.4		89.8	94.6		82.3	90.2
	ResNet101 (256, C = 4) * [47]		94.9	97.4		90.1	94.7		79.6	88.5
	BFPN [12]	-	87.0	93.0	-	82.0	91.0	-	-	-
	OR-Skip-Net [56]	98.92	96.14	98.02	98.94	88.8	94.01	99.7	83.79	91.07
	X-RayNet-1 (proposed method)	99.06	96.65	98.29	99.16	90.99	95.22	99.8	88.72	93.94
	X-RayNet-2 (proposed method)	98.93	96.14	98.02	98.96	89.30	94.25	99.8	86.65	92.73

~ represents the experiment without data augmentation. * ResNet50 and ResNet101 are used as the backbone network for Mask-RCNN; 512/ 256 shows that the input image size is (512 × 512)/(256 × 256), where C represents the number of the convolutional layer in the mask prediction branch of Mask-RCNN by Wang et al. [47]. ACC means accuracy, J shows jaccard, and D means dice score.

**Table 4:** Accuracies of X-RayNet and other methods for the Montgomery County (MC) dataset (unit: %).

Type	Method	Acc	J	D
Handcrafted local feature-based methods	Candemir et al. [25]	-	94.1	96.0
	Peng et al. [2]	97.0	-	-
	Vajda et al. [64] *	69.0	-	-
Learned/deep feature-based methods	Souza et al. [43]	96.97	88.07	96.97
	Feature selection with BN [65] *	77.0	-	-
	Feature selection with MLP [65] *	79.0	-	-
	Feature selection with RF [65] *	81.0	-	-
	Feature selection and Vote [65] *	83.0	-	-
	Bayesian feature pyramid network [12]	-	87.0	93.0
	X-RayNet-1 (proposed method)	99.11	96.36	98.14
	X-Ray-Net-2 (proposed method)	98.72	94.96	97.40

* The results for [64] and [65] are taken from [2]. BN, batch normalization; MLP means multi layer perceptron, and RF shows random forest. ACC means accuracy, J shows jaccard, and D means dice score.

**Table 5:** Accuracies of X-RayNet and other methods for the Shenzhen X-ray set (SC) dataset (unit: %).

Type	Method	Acc	J	D
Handcrafted local feature-based methods	Peng et al. [2]	97.0	-	-
	Vajda et al. [64] *	92.0	-	-
Learned/deep feature-based methods	Feature selection with BN [65] *	81.0	-	-
	Feature selection with MLP [65] *	88.0	-	-
	Feature selection with RF [65] *	89.0	-	-
	Feature selection and Vote [65] *	91.0	-	-
	Bayesian feature pyramid network [12]	-	87.0	93.0
	X-RayNet-1 (proposed method)	97.70	91.82	95.64
	X-Ray-Net-2 (proposed method)	97.32	90.56	95.0

* The results for [64] and [65] are taken from [2]. ACC means accuracy, J shows jaccard, and D means dice score.

**Table 6:** Accuracies of X-RayNet trained on MC and tested on the SC dataset and vice versa (unit: %).

Method	Train	Test	Acc	J	D
X-RayNet-1	MC	SC	96.27	87.74	93.24
X-RayNet-1	SC	MC	98.10	92.52	96.06

## Appendix A

**Table A1:** X-RayNet encoder with residual mesh and feature map size of each of the following: EB, EC, OIS, IIS, and pool (indicating encoder block, encoder convolution, outer identity stream, inner identity stream, and pooling layer, respectively). The layer with “**” denotes that the layer includes batch normalization (BN) and the ReLU unit, where “*” indicates that only BN is included with the layer. The table is based on an input image size of 447 × 447 × 3.

Block	Name/Size	Number of Filters	Output Feature Map Size (Width × Height × Number of Channels)	Number of Trainable Parameters (EC + BN)
**EB-1**	EC-1_1 **/3 × 3 × 3 To decoder (OIS-1) and E-Add-1	64	350 × 350 × 64	1792 + 128
	EC-1_2 /3 × 3 × 64	64		36,928
	E-Add-1 (EC-1_1 + EC-1_2) using IIS			-
	BN + ReLU			128
**Pool-1**	Pool-1/2 × 2 To decoder (OIS-2)	-	175 × 175 × 64	-
**EB-2**	EC-2_1 **/3 × 3 × 64 To E-Add-2	128	175 × 175 × 128	73,856 + 256
	EC-2_2 */3 × 3 × 128	128		147,584
	E-Add-2 (EC-2_1 + EC-2_2) using IIS	-		-
	BN + ReLU			256
**Pool-2**	* Pool-2/2 × 2 To decoder (OIS-3)	-	87 × 87 × 128	-
**EB-3**	EC-3_1 **/3 × 3 × 128 To E-Add-3	256	87 × 87 × 256	295,168 + 512
	EC-3_2 /3 × 3 × 256	256		590,080 + 512
	E-Add-3 (EC-3_1 + EC-3_2) using IIS	-		-
	BN + ReLU			
**Pool-3**	* Pool-3/2 × 2 To decoder (OIS-4)	-	43 × 43 × 256	-
**EB-4**	EC-4_1 **/3 × 3 × 256 To E-Add-4	512	43 × 43 × 512	1,180,160 + 1024
	EC-4_2 */3 × 3 × 512	512		2,359,808
	E-Add-4 (EC-4_1 + EC-4_2) using IIS	-		-
	BN + ReLU			1024
**Pool-4**	* Pool-4/2 × 2	-	21× 21 × 512	-

**Table A2:** X-RayNet decoder with residual mesh and feature map size of each of the following: DB, DC, OIS, INIS and unpool (indicating decoder block, decoder convolution, OIS, inner nonidentity stream, and unpooling layer, respectively). The layer with “**” denotes that the layer includes batch normalization (BN) and the ReLU unit, where “*” indicates that only BN is included with the layer; “^” shows that the path comes from the encoder corresponding block using the OIS (OIS-1 to OIS-4), where MConv represents the last convolutional layer that generates the class masks. The table is based on an input image size of 350 × 350 × 3.

Block	Name/Size	Number of Filters	Output Feature Map Size (Width × Height × Number of Channels)	Number of Trainable Parameters (DCon + BN)
**Unpool-4**	Unpool-4	-	43 × 43 × 512	-
**DB-4**	DCon-4_2 **/3 × 3 × 512	512		2,359,808 + 1024
	INIS-4 */1 × 1 × 512	256	43 × 43 × 256	131,328 + 512
	DCon-4_1 */3 × 3 × 512	256		1,179,904
	Add-5 (DCon-4_2 + INIS-4 * + Pool-3^)	-		-
	BN + ReLU			512
**Unpool-3**	* Unpool-3	-	87 × 87 × 256	-
**DB-3**	DCon-3_2 **/3 × 3 × 256	256		590,080 + 512
	INIS -3 */1 × 1 × 256	128	87 × 87 × 128	32,896 + 256
	DCon-3_1 **/3 × 3 × 256	128		295,040
	Add-6 (DCon-3_2 + INIS-3 * + Pool-2^)	-		-
	BN + ReLU			256
**Unpool-2**	* Unpool-2	-	175 × 175 × 128	-
**DB-2**	DCon-2_2 **/3 × 3 × 128	128		147,584 + 256
	INIS -2 */1 × 1 × 128	64	175 × 175 × 64	8256 + 128
	DCon-2_1 **/3 × 3 × 128	64		73,792
	Add-7 (DCon-2_2 + INIS-2 * + Pool-1^)	-		-
	BN + ReLU			128
**Unpool-1**	* Unpool-1	-	350 × 350 × 64	-
**DB-1**	DConv-1_2 **/3 × 3 × 64	64		36,928 + 128
	DConv-1_1 /3 × 3 × 64	2		36,928
	Add-8 (DCon-1_1 + DConv-1_2 + EC-1_1^)			-
	MConv **/3 × 3 × 64	4	350 × 350 × 4	2308
	BN + ReLU			8
